# Understanding Blood versus Blond Orange Consumption: A Cross-Cultural Study in Four Countries

**DOI:** 10.3390/foods11172686

**Published:** 2022-09-02

**Authors:** Adrián Giménez-Sanchis, Kui Zhong, Aurora Pintor, Vittorio Farina, Cristina Besada

**Affiliations:** 1 Sensory and Consumer Science Research Group, Postharvest Department, Valencian Institute of Agricultural Research Crta Moncada-Náquera km. 4.5, 46113 Valencia, Spain; 2 Food and Agriculture Standardization Institute, China National Institute of Standardization, Beijing 100191, China; 3 Biotechnology Department, Universidad Autónoma Metropolitana Iztapalapa, Av. San Rafael Atlixco #186, Mexico City 09340, Mexico; 4 Department of Agricultural, Food and Forest Sciences (SAAF), Università degli Studi di Palermo, Viale delle Scienze, 90128 Palermo, Italy

**Keywords:** consumption contexts, barriers, facilitators, expectations, purchase intention, organoleptic properties

## Abstract

Understanding consumer perceptions and attitudes to specific fruit is key information for not only increasing fruit consumption, but also for marketing reasons. It may also give clues to breeders to set quality objectives. This study explores different aspects that help to explain blood vs. blond orange consumption: availability and consumption habit, satisfaction attributes, facilitators and consumption barriers, consumption contexts, expectations and purchase intention. The study was conducted in China, Mexico, Spain and Italy, where citrus fruit consumers were invited to respond an online questionnaire. Our results revealed Italy as the country with the highest availability and consumption of blood oranges, followed by China, Mexico and Spain. “Liking” and “healthy properties” were the most important reasons for consumption irrespectively of orange type, but certain differences among countries were detected in secondary reasons. In all the countries, “juicy” was the most relevant attribute for consumer satisfaction, followed by flavour/taste attributes. “Aromatic” and “unfibrous” were substantial requirements for Italians and Chinese, while Spaniards attached importance to the blood oranges colour. Regarding consumption contexts, “eat with salt or chilly powder” was specific for Mexico, while “to improve health”, “as a gift” or “at a restaurant” were contexts mainly cited in China. Despite taste preferences for other fruit being the main consumption barrier in all the countries for both orange types, the relevance of other barriers depended on culture and orange type. Mexican participants seemed to take a more neophobic attitude to blood oranges, while “inconvenient” was reported as a barrier for consuming blond ones in Spain and China. We conclude that blond and blood oranges can co-exist on markets at a high consumption rate, as in Italy. Specific interventions are needed in other countries because consumer attitudes to oranges, mainly blood ones, depend on culture.

## 1. Introduction

Among fruit species, citrus fruit represents the major fruit tree crop in the world, with 10 million of harvested hectares and global estimated production close to 158 million tons [1]. In quantity terms, orange (*Citrus sinensis* (L.) Osbeck) production is the most important of the main citrus species (close to 4 million of harvested hectares and 75.5 million tons of production). However, orange production has remained relative stable in the last 10 years, while production of other citrus fruit such as mandarins has remarkably increased.

One strategy recently adopted by the citrus industry to build momentum on the oranges market is the promotion of blood oranges. Consumers’ awareness about the effect of food on their health has substantially increased in the last decade [2,3], and in these oranges with flashy pigmented pulp, the citrus industry has found an opportunity to respond to consumer demand for healthier food. Blood orange varieties are highlighted, in relation to blond ones, for their high content of the bioactive compound anthocyanin, which confers fruit red pulp colouration [4,5]. Anthocyanin may help to prevent diabetes, heart disease and viral activity, and has been shown to have promising anticancer properties [6].

Traditionally, blood oranges have been mainly cultivated in Sicily, where environmental conditions promote fruit colouration [7], and have been commercialised all over Italy, where they are well-placed on markets. Currently, the popularity of these oranges is growing worldwide [8]. Breeding programmes that focus on blood varieties have been established [9,10], and the number of research works about their physico-chemical and sensory properties is growing all around the world [8,11,12,13,14,15].

Consumers’ experience of orange purchases must be inevitably affected by the expansion of the offer with red-pigmented varieties that, in certain countries, may be a novelty. Two recent studies have approached consumers’ acceptance of blond and blood oranges based on organoleptic properties. Ávila et al. [14] after performing an acceptance test with 100 Brazilian volunteers reported strong acceptance and positive perception of consumers in relation to the most sensory attributes of blood oranges. According to the results from a study including 224 Californian people (152 adults and 72 children) [8], lighter coloured varieties, such as “Cara Cara”, “Tarocco”, “Boukhobza” and “Shahani”, were liked by the majority of consumers, and were low in less desirable sensory characteristics, such as bitterness and sourness, than the most pigmented ones. 

Despite the indubitable value of sensory studies, many consumer experience aspects cannot be measured or described by them [16]. Thus, aspects other than sensory perception that affect consumers’ consumption experience need to be investigated. Along these lines, after performing a word association test with Brazilian consumers, Ávila et al. [14] reported that they showed mainly positive attitudes to blood oranges. In a study in which Californian adults and children gave their views of oranges versus mandarins, consumers showed an interest in varieties that can be identified by their aspect, such as the blood orange Cara Cara [16]. 

Overall, the few existing works on this matter show that blood oranges represent a promising category for the citrus industry and warrant further investigation [8]. In the current context of markets internationalisation, to maintain the blond orange market position and to successfully introduce blood varieties, a better understanding of their consumption is needed. Therefore, it is necessary to investigate not only consumer expectations with regard to quality, but also other aspects of consumption experiences, such as when, why and why not [17] each orange type is consumed.

It is also important to bear in mind that all these aspects of consumers responses are strongly influenced by the cultural background [18,19,20] which may, in some cases, be linked with the level of familiarity with similar products [21]. Cross-cultural studies allow differing patterns among consumer populations to be identified, which leads to a better understanding of global market sand can help breeders and industry to adapt their product depending on its destination market [19]. In other commodities, acquiring knowledge from consumers about different aspects associated with consumption has proven to be key for production purposes and developing communication strategies [22,23,24,25]. 

Studying consumer attitudes to a product, while covering a range of different aspects, usually implies the need to combine different types of questions in the same questionnaire. This is the case of using multiple-choice questions and Likert scales, which are useful tools to collect a range of information in cross-cultural studies [26]. Multiple-choice questions allow participants to check multiple responses for a list of options and have been used to evaluate, among others, consumption contexts [27], label information required by consumers [28] or consumer behaviour with regard to food storage at home [29]. Likert scales are appropriate for evaluating intensities of different aspects, such as sustainability perception [30], food neophobia [31] or the importance of food characteristics for choices in different contexts [32].

With all this in mind, and to gain a deeper understanding of consumers’ consumption of blond and blood oranges, this study investigates their market availability, the importance of different sensory attributes for consumer satisfaction, and consumption contexts and consumption barriers in four different countries: China, Mexico, Spain and Italy. Of these countries, China, Mexico and Spain (each one situated in a different continent) are the world’s first, fourth and sixth largest citrus fruit producer countries, respectively. Italy, located in Europe such as Spain, occupies thirteenth position, but it stands out for its long-standing tradition in blood orange production and consumption. 

## 2. Materials and Methods

### 2.1. Consumer Sample

Six hundred and five people of each nationality, all of legal age, participated in this study. Sampling was carried by members of all the four participating institutions (Valencian Institute of Agricultural Research from Spain, the Università degli Studi di Palermo from Italy, the Universidad Autónoma Metropolitana from Mexico and the China National Institute of Standardization). Participants were recruited by several approaches: street surveys using a tablet device or a QR with a link to the survey, and student mailing lists of the four institutions. Snowball recruitment (word-of-mouth) was also adopted using interpersonal relations and connections among consumers to reach a large number of participants. Thus, participants were requested to invite family, friends and/or colleagues to participate by forwarding the online survey link. Only the people who reported consuming citrus fruit (whether fresh or otherwise) at least once a month during the commercial season were invited to participate. Consent indicating voluntary participation was obtained from each participant at the beginning of the questionnaire. They were shown the following statement: “If you agree to participate and us employing your answers for this study, please click next”. 

Table 1 shows the demographic information of each nationality. The recruitment of participants was carried out with the aim to obtain a similar percentage of responses from young adults (18–30 years old), middle-aged adults (31–50 years old) and late-aged adults (more than 50 years old). Moreover, the proportion of men and women was similar in all four countries, with a percentage of women between 58–60%. In educational attainment terms, the proportion of respondents with a bachelor’s degree was higher among the four nationalities, and was the equivalent to 65–74%.

### 2.2. Survey Design

This study was based on an online questionnaire via the Gloogle Forms platform (Google LLC, Mountain View, CA, USA) in the case of Spain, Italy and Mexico, and via Wenjuanxing Changsha Ranxing Information Technology Co., Ltd, Chinain the case of China. The online survey included different sections that were answered by the participants in relation to blond and blood oranges.

In order to contextualise the participants, pictures of blond and blood oranges were shown to them before they answered the questions (Figure S1). To avoid possible bias due to the presentation order, half the consumers were firstly shown blond oranges and their corresponding questions, while the other half started the questionnaire with blood oranges.

Section 1. Availability and consumption

In the first section, consumers were asked about the availability of oranges in their usual shopping place and their consumption habit by means of a multiple-choice question. The provided options were: (1) “They are available where I usually buy fruit. I usually consume them”; (2) “They are available where I usually buy fruit, but I don’t usually consume them”; (3) “They are not available where I usually buy fruit”.

**Figure 1:**
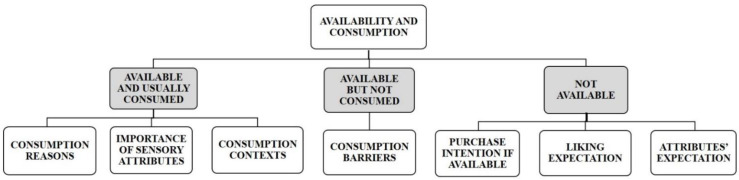
Scheme of the main questionnaire sections.

The participants were then guided to the second questionnaire section, which depended on the response they gave to the initial question, and according to the scheme shown in Figure 1 and Table S1.

Section 2A. Reasons to consume, sensory attributes’ importance and consumption context

The consumers who stated that blond/blood oranges are available in their shopping place and they usually buy them, were then requested to answer three questions in which we investigated the reasons to consume, sensory attributes’ importance and the consumption contexts of the two orange types.

In order to know the main reasons for consuming blond and blood oranges, the participants were asked the following question: “What are your main reasons for consuming these oranges (blond oranges/blood oranges)? Check all that apply”. The multiple-choice list of the possible answers included: “I like them a lot”, “Because of their healthy properties”, “Because of their affordable price compared to other fruit”, “They last longer than other fruit” and “They are more convenient (easier to carry, to eat, etc.) than other fruit”. The participants had the possibility of adding another reason for consuming them with the option “others”.

After that, this group of participants was requested to answer the following question: “Imagine that you are eating the oranges (blond orange/blood orange) that you have just bought. How important are the following characteristics to buy them again?” They had to rate the importance of certain sensory attributes to buy the fruit again using a 7-point Likert scale, where 1 = not important at all, 7 = very important [33]. The attributes to evaluate were: “being seedless”, “being easy to peel”, “not being messy when eating”, “being sweet”, “a balance between sweetness and acidity”, “being aromatic”, “having intense flavour”, “being juicy”, “not being fibrous (easy to chew)” and “having an intense pulp colour”.

After rating the sensory properties, the participants were asked about the main consumption contexts by means of the following multiple-choice question: “In which of these contexts do you consume or do you think it is appropriate to consume these oranges (blond oranges/blood oranges)? Check all that apply”. The provided options were: “when I am trying to lose weight”, “as a gift”, “when I want to improve my health”, “as part of breakfast”, “as a snack at home”, “as a snack when I’m not at home (at work, university, travelling, at a picnic, etc.)”, “In a school lunchbox”, “as dessert for lunch or dinner at home”, “at a restaurant”, “to make fresh juice or smoothies”, “to make cocktails”, “as a cooking ingredient (for salad, baking, sauces, etc.)”, “to eat with salt or chilli powder” or “to eat with sugar”. They also had the possibility of adding another possible consumption context through the option “others”.

Section 2B. Consumption barriers

The participants who indicated that they do not usually consume fruit, even though it is available in their regular purchase place, were requested to indicate their main purchase barriers by answering the multiple-choice question that follows: “What are the main reasons for you not consuming this kind of oranges (blond oranges/blood oranges)? Check all that apply”. The available options were: “I prefer the taste of other fruit”, “I have never tasted it”, “I have tasted it, but don’t like it”, ‘It’s too expensive”, “I think they are not natural”, “I don’t like the way it looks”, “I don’t know how to eat/prepare it” and “I think they are not safe”. They had the possibility of adding another reason through the option “others”.

Section 2C. Liking expectations and purchase intention

Finally, the consumers for whom fruit is not available in their shopping place had to answer a different set of questions.

They were asked about their expected liking and purchase intention if fruit were available. The questions were formulated as follows: “If these oranges were available where you usually buy fruit, would you buy them?” and “How much do you expect to like this fruit?”. The scales to respond were on a 5-point scale ranging from 1—“I definitely would not buy” to 5—“I definitely would buy” and on a 7-point hedonic scale where 1 = I will dislike it very much and 7 = “I will like it very much”, respectively.

Further, to know their perception and expectations about fruit, they were requested to answer the next multiple-choice question: “What do you think about this fruit? Check all that apply”. The response options were the following: “it’s very healthy”, “it’s natural”, “it’s not safe for eating”, “it’s appealing”, “it’s a new fruit”, “it comes from another country”, “taste will be similar to other citrus fruit”, “taste will be surprising”, “it will be sweet”, “it will be acid”, “it will be bitter” and “it will be more expensive than other fruit”. Participants had the option to add more reasons through the option “others”.

The lists of options presented to the participants in the different multiple-choice questions (reason for consumption, sensory attributes, barriers, contexts and expectations) were preliminarily created by the Spanish research group based on the previous literature about citrus and other fruit [20,23,25,27,28,32,34,35,36,37,38,39,40,41,42] (Table S2). The initial lists, which covered the most relevant options according to the existing literature, were completed by performing a test run with 10 consumers to add any missing option relevant for them. The lists generated in Spain were checked and completed by researchers of the other three countries and then pre-tests were carried out with the consumers from the different countries to add country-specific options. Thus, for example, in the contexts section, options “to eat with salt or chilli powder” and “as a gift” were added by request from Mexican and Chinese consumers, respectively. The presentation order of the list terms was balanced among consumers to avoid bias.

The last questionnaire section (Section 3) was about demographic data: gender, age and level of education.

The final questionnaire was originally generated in Spanish by the Spanish research group and underwent a pilot trial with consumers (*n* = 10) to check for errors, ambiguity, logical flow of questions and completion time. The questionnaire was directly sent to Mexico where it was pretested with 10 consumers.

In parallel, a professional translator translated the questionnaire into English before sending it to Italy and China. The native members of the participating research institutions with good knowledge in English translated the questionnaire into Italian and Chinese. The questionnaires in each language were then carefully assessed and back-translated into English by different researchers from each country to ensure that the meaning of the original content was maintained and the four versions were consistent with one and other. Before releasing the final questionnaire, it was pretested in Italy and China with 10 consumers.

The protocol and procedures used in this study were revised by the scientific directorate of Valencian Institute for Agricultural Research, which stated a waiver consent. All articles from the Declaration of Helsinki and the 2016/679 EU Regulation on the protection of natural persons regarding the processing of personal data and on the free movement of such data were met.

### 2.3. Statistical Analysis

To analyse the data, different non-parametrics tests were performed. The comparison of k proportion-test (procedure of Marascuilo and chi-cuadrado, *p* < 0.05) was used to compare the availability and consumption data. The two-proportion z-test (*p* < 0.05) was used to compare the frequencies of mentioning of reasons to purchase in each country depending on the orange type. The Kruskall–Wallis test followed by Dunn’s multiple comparisons test were applied to evaluate for each country and orange type the differences in importance for repurchase among attributes (*p* < 0.05).

A correspondence analysis (CA) was carried out with the percentages of participants from each country who mentioned each consumption context (of the participants who stated consuming each orange type), purchase barrier (of those who stated not consuming) and expectations (of those who stated not having orange availability) to visualise the more relevant differences among the four countries and between the two orange types. All the analyses were performed with XLStat 2019 (Addinsoft, Paris, France).

## 3. Results and Discussion

Availability has been reported as a prime factor for determining the consumption of specific fruits [43]. Thus, a question about blond and blood oranges availability in the habitual fruit shopping place and consumption habit was the starting point of our questionnaire. Then depending on the response to the initial question, the participants were guided to different sections to investigate some of the main aspects of consumer behaviour with regard to oranges: (a) reasons to consume, sensory attributes importance and consumption contexts; (b) barriers to consume; (c) expectations and purchase intention.

### 3.1. Availability and Consumption Habit

The results of the availability and consumption rate for blond and blood oranges are shown in Table 2. Blond oranges availability on markets was almost 100% in the four countries under study, except for China where 3% of the participants stated that blond oranges are not available where they usually buy fruit. The lowest percentage of consumption was also detected in this country. Seventy-four percent of the Chinese participants were habitual consumers of blond oranges. In Spain, Italy and Mexico, the consumption rate was higher than 85%.

The data about blood oranges revealed a very different picture compared to the blond ones. Italy was by far the country with the highest availability (98%) and the highest consumption rate (76%). China occupied second position for both availability (83.5%) and purchase rate (46%). In Mexico and Spain, availability was much lower because only 50% of the participants can buy blood oranges on their habitual markets. Of the four studied countries, the lowest consumption was detected by far in Spain, where only 17% usually consume blood oranges.

Our data revealed a similar pattern among countries for blond oranges, but a very different one for blood ones. The data from both kinds of oranges taken together confirmed that availability is a prime factor for the consumption of specific fruits, as previously reported by Konopacka et al. [43].

As explained in the Introduction, blood oranges are a traditional crop in Italy, which is the first growing country and the origin of these pigmented fruits [7]. Accordingly, our data showed that both availability and consumption rate were much higher in this country than in the other three. Furthermore, the data indicate that Italy represents a scenario in which blond and blood oranges naturally co-exist on markets.

### 3.2. Reasons to Consume, Sensory Attributes’ Importance and Consumption Context

#### 3.2.1. Reasons to Consume

The relevance of identifying reasons to consume (facilitators) and fruit consumption barriers is well-known but, until now, it has been mostly approached by considering fruit to be a whole group of food rather than evaluating specific commodities [41,44].

In order to identify the main facilitators for orange consumption, we asked those participants who stated that they consume them habitually about the main reasons for doing so. Our data revealed that irrespectively of country, the core drivers to consume both blond and blood oranges are liking (“I like them a lot”) and health benefits (“Because of their healthy properties”) (Table 3).

In Mexico and China, the frequency of mentioning the other three reasons was similar and ranged among 25–50%. After health benefits, the importance of reasons was as follows: price, convenience and durability. In Mexico, the percentage of consumers who cited all these three reasons was slightly higher for blond than for blood oranges. In China, only “affordable price” seemed to be slightly more important for buying blond than blood oranges.

The ranking of reasons for the Italian consumers was the same as in Mexico and China. However, it is important to highlight that the percentage of consumers who cited price, convenience and durability reasons was much lower in Italy. This result revealed that liking and healthy benefits are indisputably the most important reasons for Italians buying oranges. In this study, the data from Italians were obtained mainly but not exclusively from consumers living in a citrus-producing region (Sicily). The herein identification of liking and healthy benefits as core drivers for consumption is in accordance with previous studies performed in different areas around Italy [36,38,39]. Thus, these studies revealed the “healthy identity” of citrus fruit and its pleasant taste as highly relevant attributes for Italians, while affordable price and ease of peeling were not relevant.

Finally, a different ranking order of the secondary reasons was detected in Spain, where consumers attached more importance to oranges’ durability than in the other countries. In this country, the percentage of participants who selected all five reasons was higher for blond than for blood oranges. This difference was especially evident for “affordable price”, which is explained by the fact that blood oranges in Spain are generally more expensive on markets that blond ones.

The results of this study support previous findings that have demonstrated the importance of healthiness and taste for consumer food choice [36,38,39,45]. Along these lines, in a cross-cultural study that included several European countries, Onwezen and Bartels, [34] identified four consumer segments based on their reasons for choosing fruit and reported natural and healthy as the main reasons for one of the groups, while healthy and taste were the most important reasons for fruit choice in three of the four identified profiles.

Despite the consensus among countries about identifying liking and health as prime reasons for blond and blood orange consumption, our results show relevant differences among countries with regard to the other investigated reasons. So, the number of Italian consumers for whom price, convenience and durability were the reasons for choosing oranges was significantly smaller than in the other countries. Familiarity with the product did not seem to explain such differences because the result was equally observed for blond and blood oranges, while difference in familiarity among Italy and the other countries was detected only for blood ones. Therefore, the importance of the different reasons for food choice seems to be influenced by cultural factors beyond familiarity, as previously reported by Nielsen et al. [46] and Prescott et al. [47].

#### 3.2.2. Sensory Attributes’ Importance for Repurchase

One of the reasons for lower than recommended fruit consumption is that the fruit market does not always meet consumer expectations [34]. Therefore, providing markets with fruit that satisfy consumers could help with the challenge of increasing a global population’s fruit consumption. To fulfil this objective, it is necessary to understand consumer expectations in terms of sensory properties while bearing in mind that such expectations may differ among populations from different countries [19].

Consumer satisfaction is key for repurchasing a product [48]. Thus, in this study, we asked consumers to rate the importance of finding different sensory properties in each orange type in their repurchasing intention. The results of this question are shown in Table 4, in which the attributes are ranked according to their importance score. Mean values of importance scores are shown in detail in Table S3. As we can see, irrespectively of country and orange type, consumers agreed that “juicy” was the most important attribute for their satisfaction.

Spaniards and Mexicans agreed in considering that three attributes related to flavour/taste (“intense flavour”, “sweet” and “balanced sweet-acid”) were the most important attributes after “juicy” and reported “being aromatic” and “not being fibrous” as the next most important attributes. However, the Italian and Chinese found the “being aromatic” attribute to be equally important to those linked with taste/flavour. Smell has been previously reported as a relevant attribute for Italian consumers [39]. Furthermore, for Chinese consumers the texture attribute “unfibrous” was much more relevant than in other countries and was equally important as the flavour/taste and “aromatic” attributes.

“Being easy to peel”, “being seedless” and “not being messy when eating” are attributes particularly linked with fruit convenience [16,49,50]. In general, these three attributes were ranked together and were less important to consumers than the other attributes.

The attribute in which the consumers from the different countries differed the most in importance terms was “having an intense pulp colour”. In Italy, Mexico and China, there were no differences between blond and blood oranges. For the Chinese consumers, it was the least important attribute, while for the Italian and Mexican consumers, it was more important than the group of convenience attributes (“being easy to peel”, “being seedless” and “not being messy when eating”). Spain was the only country where clear differences were observed in the importance of colour between the two orange types because the Spaniards considered colour to be more important than the convenience attributes in blood oranges, but not in blond oranges.

These results fall in line with recent studies on mandarin fruit in which attributes, such as “juicy”, “sweet” and “sour”, were identified as drivers of liking for Spanish and Mexican consumers [20,40]. By comparing the results of these two studies, which focused on mandarins, to the results herein obtained, it seems that “easy to peeling” is a more relevant attribute for mandarins than for oranges. Along these lines, after investigating what kind of sensory information consumers would like to receive when buying different types of fruit, Fernández-Serrano et al. [28] found that the juicy, flavour/taste related attributes and easy peeling were the most relevant information for citrus fruit as a group.

Drivers of liking have been traditionally identified by means of sensory tests in which liking scores given by consumers are related to the sensory attributes perceived by a trained panel or consumers themselves after tasting fruit [40,42,51]. In this study, evaluating the importance of different attributes by means of an online questionnaire filled in by consumers without testing any sample led to similar results to existing studies with sensory tests. Our approach based on an online questionnaire is very similar to that followed by Galmarini et al. [19] to evaluate the attributes that define apple quality for Argentinean and French consumers. As in our case, those authors found some differences among countries, mainly in the importance of visual aspects, because they were more important for Argentinean consumers, while the French ones showed more interest in taste-related attributes.

To go one step further, we investigated if the satisfaction attributes depended on the gender and age of the participants. Results from ANOVA analysis are shown in Figures S2–S5. Regarding gender influence (Figures S2 and S3), it is worth to comment that a gender effect was detected in the importance that Italians and Mexicans gave to the three convenience attributes (easily peeling, no messy and seedless). Curiously enough, women from Italy gave less importance than men to these three attributes, while an opposite pattern was observed in Mexico.

As far as the participants age is concerned, the main effect was detected in Spain, where the youngest consumers (13–30 years old) gave lower importance scores to all sensory attributes except the flavour intensity and juicy (Figures S4 and S5).

#### 3.2.3. Consumption Contexts

The role of consumption contexts in purchase is perhaps less obvious than that of facilitators or sensory properties, but there are reports showing that the final decision to buy or consume a particular food depends as much on the anticipated usage context as it does on intrinsic product properties [52]. So recent studies on fruit have paid attention to it [27,37].

For both orange types, the more frequently mentioned contexts for the Italian, Spanish and Mexican consumers were “as a dessert for lunch or dinner at home”, “as part of breakfast”, “as a snack at home” and “as a snack when I’m not at home (at work, university, travelling, at a picnic, etc.)” and “to make fresh juice or smoothies” (Table S4). For Chinese people, these five contexts were relevant, but “when I want to improve my health” was also among the most cited contexts and occupied second position.

**Figure 2:**
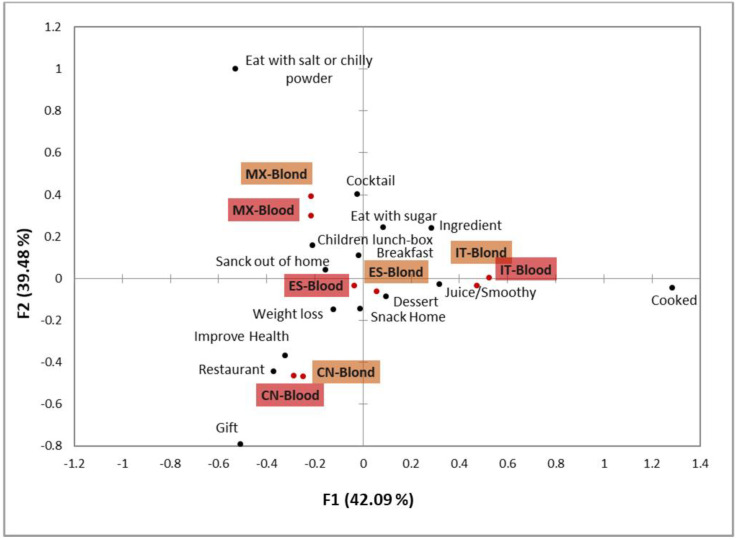
Correspondence analysis for the consumption contexts. The data used to perform the analysis were the percentage of participants (of those who stated consuming each orange type habitually) who selected each context. ES—Spain, IT—Italy, CN—China and MX—Mexico.

For a profounder evaluation of the main similarities and differences among countries, a CA was performed (Figure 2). The first result of this analysis to highlight is that for the four countries, blond and blood oranges were very close in the CA space. This indicates that consumption contexts did not depend on oranges being blond or blood.

The Spain and Italy samples were allocated very close to one and other, and very close to the origin on the first dimension and with negative values on the second one. The contexts “as part of breakfast”, “as a dessert for lunch”, “to make fresh juice or smoothies”, “as a cooking ingredient” and “to eat with sugar” were characteristics of these two countries compared to Mexico and China. Consuming oranges or orange juice as part of the breakfast has been previously reported as a main consumption context in Spain and Italy [27,39].

The Mexico samples gave positive values on the second coordinate and negative values on the first one. This position on the CA plot was explained by the contexts “to eat with salt or chilli powder” and “to make cocktails”, which were mainly mentioned in this country compared to the other three. Finally, consumption of oranges in China was much more frequent “as a gift”, “when I am trying to lose weight”, “at a restaurant” and “when I want to improve my health”.

Differences among countries in consumption contexts were more marked than those detected in facilitators for purchase or sensory attributes’ importance. Thus, some contexts seemed specific for certain countries. This was the case of “with salt and chilli powder” in Mexico and “as a gift” in China.

According to Gutierrez and Simon [53], the main reason why spicy food was more frequently consumed in hot climate countries such as Mexico in the past is because of the antibacterial characteristics of spices that contribute to people’s health. However, these authors suggest that presently, people like spicy food not for its historical antimicrobial properties, but because it is rooted in their culture.

Tradition seems to also be linked with the Chinese habit of giving fruit as a gift. According to Vasil’eva and Chibisova [54], to offer a live orange tree or its fruit as a gift is an ancient tradition in China, meaning a wish of happiness, wealth and success. In the same line, a traditional Chinese value is to regard food as “medicine” [47], which would explain why consumption contexts such as “when I am trying to lose weight” and “when I want to improve my health” were more frequent in China than in Mexico or European countries.

### 3.3. Consumption Barriers

Barriers, as defined by Yeh, et al. [55], are factors that inhibit consumption. Identifying barriers is important for designing behaviour change interventions to increase the consumption of specific food [56], and for industry to anticipate potential obstacles and mitigate their impact.

Table S5 shows the percentage of consumers who cited each consumption barrier. We can observe that the main barrier stated by all consumers irrespectively of country and orange type was “I prefer other fruit”. In Spain, Italy and Mexico, this barrier was by far the most cited one. However, in China, it was cited by fewer consumers, and was closely followed by the “Too expensive” barrier, mainly for blood oranges. Previous studies investigating fruit consumption barriers as a whole group of food have demonstrated that taste preferences for other products are among the common self-reported barriers to eat fruit [57]. Moreover, in a recent review about fruit and vegetable consumption in east and southeast Asia, Cheung et al. [58] found that financial concerns stemming from high prices of fruit and vegetable are major barriers for their consumption.

It is important to clarify that “Because it is inconvenient” was not initially included in the list of barriers for the participants to check. However, as it was indicated by means of the “Others” option by a significant percentage of respondents from China and Spain, we decided to include it in the results.

**Figure 3:**
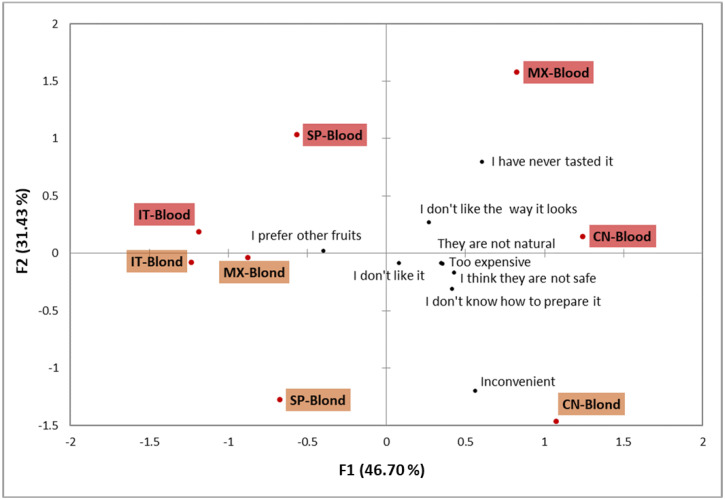
Correspondence analysis for the consumption barriers. The data used to perform the analysis were the percentage of participants (of those who stated not consuming each orange type habitually) who selected each barrier. ES—Spain, IT—Italy, CN—China and MX—Mexico.

Despite taste preferences being the most cited barrier in all the countries, the CA plot revealed certain differences in the relative importance of this and other barriers depending on culture and orange type (Figure 3). Thus, the samples for which taste preferences were by far the most important barrier were allocated on the left of the CA plot, while those samples for which the relative importance of this barrier was not as marked were allocated to the right of the space.

Italy was the only country for which blond and blood oranges allocation on the CA plot was very close. Thus, taste preferences were indisputably the main barrier for both orange types in Italy.

Samples from Spain were also allocated in the left area of the plot but were separated along the second dimension. While “inconvenient” was a barrier for blond oranges, “I have never tasted” and “I don’t like the way it looks” were the reasons reported by Spaniards to not consume blood ones.

Mexico was the country with the greatest distance between blond and blood oranges. In fact, each one was located on one side of the plot. Taste preferences were clearly the most relevant barrier for Mexicans to consume blond oranges, which was on the same line as in Italy. The blood orange sample from Mexico was allocated to the right of the plot together with both samples from China. Comparatively to other samples, the barrier “I prefer other fruits” was less frequently cited in these three cases. Barriers such as “it is too expensive”, “I think they are not safe”, “I don’t know how to prepare it”, “They are not natural”, “I have never tasted” and “I don’t like the way it looks” had greater relative importance in China and Mexico than in Italy and Spain, and mainly for blood oranges. “Inconvenient” was identified as a relevant barrier for blond orange consumption in China, as it was in Spain.

It is clear from the CA plot that familiarity had a marked influence on barriers for blood orange consumption. Thus, while taste preferences were the major barrier in Italy, “I have never tasted” and “I don’t like the way it looks” were relevant barriers shared by the other three countries. In line with this, after evaluating consumers’ response to carrots with atypical colours, Schifferstein et al. [59] reported that consumers gave lower attractiveness ratings to the unfamiliar variants and suggested that at least at first glance consumers may be reluctant to try them. These authors also found that consumers may perceive unfamiliar coloured products as unnatural/artificial, which was the case of the Chinese and Mexicans in this study. This perception is related to the “food neophobia” concept, which is defined as the reluctance to eat unfamiliar food [60]. This phenomenon has been associated with the omnivore’s dilemma while searching for food: approaching novel foods or protecting oneself from potentially poisonous food, thus restricting one’s diet [61,62]. This may also explain the perception of novel food as not safe.

With regard blond oranges, the relevance of “inconvenient” as a barrier in Spain and China is noteworthy. As previously mentioned, this barrier was included by the participants from these two countries by means of the “Others” option. Some participants wrote down “more inconvenient than other citrus fruit”, which gave us a clue to understand the cultural specificity of this barrier. In a recent study in which consumers were asked to group different fruit types based on characteristics that influenced their consumption context, oranges were included in the “Cutlery-needed fruit” (one-serving fruit that needs cutlery for the eating process), while mandarins were included in the “Easy-to-peel fruit” (one-serving fruit, easy to transport, that does not need to be peeled or is handy to peel) [27]. This study clearly showed that consumers consider mandarins a more convenient citrus fruit than oranges. According to FAO statistics [1], mandarin production is much higher in China and Spain than in Italy and Mexico. Thus, our hypothesis in this regard is that Chinese and Spaniards are more familiar with mandarins, and they consider oranges a less convenient fruit because they instinctively compared it to mandarins.

### 3.4. Purchase Intention and Consumer Expectations

Schifferstein et al. [59] reported that increasing the vegetables offer with varieties with unfamiliar aesthetic characteristics to the naïve consumer may lead them to uncertainty about what qualities and taste experiences to expect. Our objective with this questionnaire section was to investigate the expectations of unfamiliar consumers with the product due to lack of access to it on markets.

As explained in Section 3.1, orange availability was almost 100% for blond oranges in the four studied countries and for blood oranges in Italy. Therefore, the results shown in this section focused on blood oranges and on Spanish, Mexican and Chinese consumers. Other than expectations, we asked them about their purchasing intention if blood oranges were available in their habitual shopping place and how much they expected to like these oranges.

**Figure 4:**
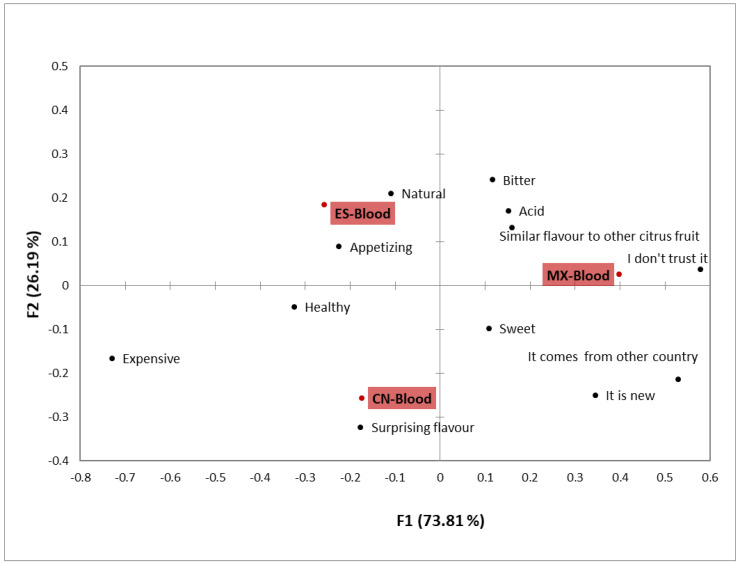
Correspondence analysis for consumers’ expectations. The data used to perform the analysis were the percentage of participants (of those who stated that blood oranges are not available in their habitual shopping place) who selected each expectation. ES—Spain, CN—China and MX—Mexico.

Figure 4 shows the distribution of blood orange expectations depending on the country. Mexican consumers defined blood oranges as a new product from another country and were somewhat suspicious of it (“I don’t trust it”). According to Salgado et al. [63], a strong attachment to their traditions is one of the main reasons for Mexican consumers rejecting new foods, especially adults.

Chinese people expected blood oranges to have a surprising flavour, while Spaniards reported mostly expectations not directly linked with sensory properties. They expected blood oranges to be natural, appetising and healthy. Both the Spanish and Chinese’ consumers expected blood oranges to be an expensive product.

Despite the differences found in attribute expectations, when the participants were directly asked about how much they expected to like blood oranges, the expected liking was similar in Spain, China and Mexico, with values close to six (data not shown). Certain differences were, however, detected in purchasing intention. Thus, the percentage of participants who stated that they were willing to buy fruit if it was available on the market was 40% in Spain, and 60% in China and Mexico (Figure S6).

Therefore, Spain was not only the country with the lowest consumption rate of blood oranges (Table 1), but it was also where consumers had the lowest purchasing intention. A priori, “natural”, “appetizing” and “healthy”, the attributes used by Spaniards to describe blood oranges, should have a positive impact on purchasing intention. It seems, therefore, that expectations of an expensive product may be the reason behind low purchase intention.

### 3.5. Strengths and Limitations of the Study, Practical Implications and Future Perspectives

Before closing this section, it is important to discuss the weaknesses and strengths of this study, the practical relevance of the information herein presented as well as the need of future studies.

In order to compare data from the different countries, we balanced consumer samples with regard to gender, age and level of education. All of them are factors that may have an influence on consumer response [64,65]. We decided not to ask about level of income because, in previous studies, we realised that the participants are reluctant to answer this question. However, level of income may have an influence on certain responses, mainly on the frequency of mentioning consumption barriers, such as “too expensive”. Thus, we cannot be sure if it is one of the reasons why this barrier was detected mainly in China.

It would have also been interesting to include a neophobia test at the end of our questionnaire because our results revealed certain cultural differences in this regard. Thus, the fact that the barrier “I have never tasted” and the expectation “I don‘t trust it” were more frequently mentioned in Mexico suggests that Mexicans take a more neophobic attitude to blood oranges compared to Chinese and Spaniards.

One of the strengths of this study is that it responds to a need for studies to approach the commercialisation and marketing aspects of specific fruit types rather than fruit in general [66]. By exploring consumers’ perceptions of blond and blood oranges, we obtained highly and directly applicable information. So, our findings have significant implications for the marketing and export activities of the citrus industry, particularly in a globalised world. Moreover, the information herein presented is very valuable for citrus breeders around the world to produce orange varieties that respond to consumers’ quality demand.

Another aspect to highlight of this study is its cross-cultural approach. Our study corroborated that cultural background affects how food products are perceived and, thus, consumer behaviour towards it [19,67]. A better understanding of how consumers’ perceptions differ between groups will help traders to implement the most effective interventions depending on specific markets.

Our results revealed that Italy was the country where the position that blond and blood oranges occupy on the market seems ideal. Thus, both orange types co-exist on markets with a high consumption rate. The only reason why a low percentage of Italians do not consume oranges is because they have taste preferences for other fruit, which is normal and expected even in the best scenario for commercialising a specific fruit type.

Mexico and Spain were revealed as the countries with the lowest blood oranges consumption. According to our results, 60% of Mexican consumers for whom this fruit was unavailable could be potential buyers. Therefore, increasing blood oranges availability on Mexican markets can be a good intervention to increase their consumption. Moreover, the identification of “I’ve never tasted it” and “I don’t like the way it looks” as relevant consumption barriers in this country suggests that interventions focusing on promoting people to taste fruit with tasting opportunities in fairs, supermarkets or schools may have a positive effect.

Blood oranges purchase intention was lower in Spain than in Mexico, and this seems to be related to people’s expectations of expensive fruit. Thus, increasing availability in combination with promotional offers on markets may be a good option to reach out to a big group of Spanish consumers.

Blood oranges availability on Chinese markets was high with 83%. However, a high percentage of the consumers from this country stated not consuming them. Moreover, 20% of these people also stated that they did not consume blond oranges (Table 2). Thus, identifying consumption barriers would be a key result in this country. Interestingly enough, the Chinese participants pointed out “inconvenient” as a barrier for blond oranges consumption. Selling minimally processed, already peeled and segmented oranges could be a good option to overcome this barrier. As health-related characteristics of minimally processed oranges are retained during refrigerated storage [68], this product offers the convenience and health benefits required by Chinese consumers.

The relevance of unaffordable prices as a barrier in China suggests promotional offers on markets as a promising intervention to facilitate oranges consumption and to promote the blood orange varieties. Moreover, Liu et al. [69] reported that establishing a trusted certification system would improve Chinese consumers’ food safety evaluation and increase the demand for high-quality safe fruit on the Chinese domestic market. Moreover, adding claims about the “naturalness” of blood varieties and images of the product prepared to eat/drink may also have a positive impact in this country.

Other than cultural particularities, it is also worth mentioning the aspects that were common irrespectively of country. The most important ones to highlight are the main reasons for purchasing oranges, which were identified as taste preferences and healthy properties in all four countries. Therefore, using labels that claim beneficial properties, such as high vitamin C, fibre or antioxidant compounds content, are likely to catch consumers’ attention. This may be especially useful for promoting the strong points of blood oranges because these varieties are highlighted for their high content of antioxidant compounds and anthocyanins [4]. In a recent study, Tarancón et al. [70] found a link between the rind pigmentation intensity and pulp anthocyanin content of “Sanguinelli”, one of the most important blood orange varieties. These authors also reported that fruit colouration is linked with fruit sensory properties in such a way that the more intense the red pigmentation is, the more the perceived sweetness. Thus, rind colouration may become a nutritional and quality index for both marketers and consumers. Conveying this information to consumers may be especially relevant. Hence our data showed that Chinese consumers stand out for consuming fruit when they wish to improve their health (“to improve health” and “to lose weight” contexts) (Figure 2). However, they also highlighted disregarding fruit colour (Table 4). Thus, Chinese consumers must be informed about the relation between blood oranges pigmentation and their healthy properties.

Finally, our results may throw light on breeding programmes with regard to consumers’ quality requirements. In the four evaluated countries, juicy was the most important attribute for consumer satisfaction, followed by flavour/taste attributes. Varieties with an intense aroma are specially indicated for the Italian and Chinese markets. Moreover, unfibrous varieties would be very much appreciated by Chinese consumers.

Future studies should investigate to what extent the sensory properties (other than the aspect) of blood oranges differ from those of the blond ones. This information would be useful not only for breeders but also for the citrus industry for marketing purposes.

By way of conclusion, we can state that blond and blood oranges can co-exist on markets at a high consumption rate, as in Italy. To achieve such a situation in other countries, specific approaches are needed because consumers’ perception of oranges (reasons and barriers for purchasing, consumption contexts, expectations and purchase intention) depend on culture.

## Figures and Tables

**Table 1:** Demographic data of the participants from each country expressed as a percentage.

		Spain	Italy	China	Mexico
Gender	Male	37.9%	39.5%	42.3%	31.7%
	Female	62.1%	60.5%	57.7%	68.3%
Age	18–30	31.7%	20.7%	33.4%	37.2%
	31–50	37.5%	39.3%	33.4%	42.3%
	>50	30.7%	40.0%	33.2%	20.5%
Consumption	Almost every day	52.0%	52.4%	30.0%	20.2%
	2 or 3 times a week	30.4%	25.7%	29.7%	44.8%
	2 or 3 times a month	17.6%	21.8%	40.3%	35.0%
Education	Elementary	6.0%	0.2%	3.0%	4.0%
	Secondary	20.2%	29.9%	31.4%	23.8%
	Bachelor’s degree	73.8%	69.9%	65.5%	72.2%

**Table 2:** Percentage of participants from each country (ES-Spain, IT—Italy, MX—Mexico and CN—China) who stated that blond oranges/blood oranges were available in their habitual purchase place (Avail. column). The “Cons.” column indicates the percentage of them who stated consuming oranges habitually, and “Not Cons.” was the percentage who stated not consuming them.

	Blond Oranges			Blood Oranges		
	Avail.	Cons.	Not Cons.	Avail.	Cons.	Not Cons.
ES	99.2 ^b^	85.5 ^b^	13.7 ^b^	48.6 ^a^	17.2 ^a^	31.4 ^b^
IT	99.8 ^b^	91.6 ^c^	8.2 ^a^	98.0 ^c^	75.9 ^d^	22.1 ^a^
MX	99.8 ^b^	89.6 ^bc^	10.2 ^ab^	50.7 ^a^	34.2 ^b^	16.5 ^a^
CN	97.0 ^a^	73.9 ^a^	23.1 ^c^	83.5 ^b^	46.1 ^c^	37.4 ^b^

Different letters in the same column indicate significant differences among countries (comparison of k proportions-test, *p*-value = 0.05).

**Table 3:** Percentage of participants from each country (of those who stated consuming each orange type habitually) who selected each reason to consume. Conven.-Convenience. Durab.-Durability.

Spain		Italy		Mexico		China	
Blond	Blood	Blond	Blood	Blond	Blood	Blond	Blood
Liking (84.4%) *	Liking (72.1%)	Liking (87.5%)	Liking (85.4%)	Liking (83.4%)	Liking (85.0%)	Liking (74.2%)	Liking (73.7%)
Health (68.3%) *	Health (52.9%)	Health (57.8%)	Health (53.3%)	Health (64.4%)	Health (58.9%)	Health (61.2%)	Health (65.5%)
Durab. (34.9%) *	Durab. (21.2%)	Price (11.3%) *	Price (7.0%)	Price (49.3%) *	Price (39.6%)	Price (37.7%) *	Conven. (32.7%)
Price (31.9%) *	Conven. (18.3%)	Conven. (9.5%)	Conven. (6.3%)	Conven. (46.3%) *	Conven. (36.2%)	Conven. (36.3%)	Price (29.5%)
Conven. (29.6%) *	Price (8.7%)	Durab. (8.5%) *	Durab. (4.3%)	Durab. (36.2%) *	Durab. (24.2%)	Durab. (28.9%)	Durab. (24.8%)

* Indicates significant differences between blond and blood oranges (z-test, *p*-value = 0.05).

**Table 4:** Ranking of sensory attributes’ importance for consumer satisfaction after eating blond/blood oranges. Importance was scored in a 7-point scale (1—not important at all, 7—very important) by Spanish, Italian, Mexican and Chinese consumers (*n* = 605 in each country).

Spain		Italy		Mexico		China	
Blond	Blood	Blond	Blood	Blond	Blood	Blond	Blood
Juicy ^a^	Juicy ^a^	Juicy ^a^	Juicy ^a^	Juicy ^a^	Juicy ^a^	Juicy ^a^	Juicy ^a^
Flavour ^b^	Sweet ^ab^	Flavour ^a^	Flavour ^b^	Sweet ^b^	Sweet ^ab^	Sweet ^ab^	Sweet ^ab^
Balance ^b^	Balance ^ab^	Aromatic ^b^	Aromatic ^c^	Balance ^bc^	Flavour ^bc^	Unfibrous ^b^	Aromatic ^ab^
Sweet ^c^	Flavour ^ab^	Balance ^b^	Sweet ^c^	Flavour ^c^	Balance ^bc^	Aromatic ^bc^	Unfibrous ^ab^
Aromatic ^d^	Aromatic ^ab^	Sweet ^b^	Balance ^c^	Aromatic ^d^	Aromatic ^cd^	Flavour ^bc^	Balance ^ab^
Unfibrous ^d^	Colour ^bc^	Colour ^c^	Colour ^d^	Unfibrous ^d^	Unfibrous ^d^	Balance ^bc^	Flavour ^ab^
Easy Pe ^e^	Unfibrous ^c^	Unfibrous ^d^	Unfibrous ^e^	Colour ^e^	Colour ^d^	NoMessy ^c^	NoMessy ^bc^
Seedless ^e^	Easy Pe ^d^	Easy Pe ^d^	Easy Pe ^e^	Easy Pe ^f^	Easy Pe ^e^	Easy Pe ^c^	Easy Pe ^bc^
Colour ^e^	Seedless ^d^	Seedless ^e^	Seedless ^f^	NoMessy ^g^	Seedless ^f^	Seedless ^d^	Seedless ^c^
NoMessy ^f^	NoMessy ^e^	NoMessy ^f^	NoMessy ^g^	Seedless ^g^	NoMessy ^f^	Colour ^e^	Colour ^c^

Different letters in the same column indicate significant differences among attributes’ importance (Kruskall–Wallis test, *p*-value = 0.05).

## Data Availability

The data presented in this study are available on request from the corresponding author.

## Supplementary Materials

The following are available online at https://www.mdpi.com/article/10.3390/foods11172686/s1, Figure S1. Pictures of oranges shown in the questionnaire; Figures S2–S5. Effect of gender and age in attributes importance. Figure S6. Purchase intention. Table S1: Number of participants who responded each questionnaire section; Table S2. References used to create the questionnaire lists. Table S3. Ranking of attributes importance with score values. Table S4. Percentage of participants who selected each consumption context. Table S5. Percentage of participants who selected each consumption barrier.
