# Supplementary material for: Understanding Blood versus Blond Orange Consumption: A Cross-Cultural Study in Four Countries
Source: Foods. 2022 Sep 2;11(17):2686. doi: 10.3390/foods11172686 (PMC9455469; doi:10.3390/foods11172686)
Supplement: Supplementary file 1 [file foods-11-02686-s001.zip › foods-1872493-supplementary.pdf]

Supplementary Material

UNDERSTANDING BLOOD *VERSUS* BLOND ORANGE CONSUMPTION. A CROSS-CULTURAL STUDY IN FOUR COUNTRIES.

Giménez-Sanchis, A[1]., Zhong K.[2], Pintor, A.[3], Farina, V.[4], Besada, C[1]

Figure S1. Pictures of blond (A) and blood (B) oranges shown in the questionnaire.

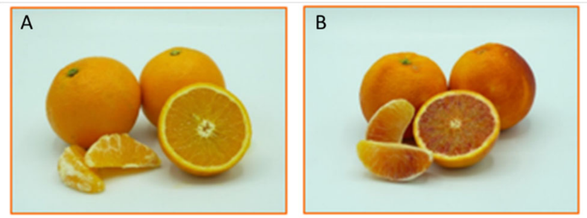

Table S1. Number of participants from each country who responded each section of the questionnaire

Availability and consumption habit	Sections	Spain		Italy		Mexico		China	
		Blond	Blood	Blond	Blood	Blond	Blood	Blond	Blood
Available and usually consumed	Reasons to consume<br>Sensory attributes' importance<br>Consumption contexts	517	104	554	459	542	207	447	279
Available but not consumed	Consumption barriers	83	190	50	134	62	100	140	226
Not available	Purchase intention if available<br>Liking expectation<br>Attributes' expectation	5	311	1	12	1	298	18	100

Table S2. List of references used to create the preliminary lists of the questionnaire.

	References
Facilitators (Reasons to consume)	Onwezen & Bartels, 2011 [34]<br>Sijtsema et al., 2012 [23]<br>Phan et al., 2016 [25]<br>Iofrida et al., 2019 [38]<br>Di Vita et al., 2020 [39]<br>Kasprzak et al. 2021 [41]<br>Ingrassia et al., 2016 [36]
Sensory attributes	Iofrida et al., 2019 [38]<br>Di Vita et al., 2020 [39]<br>Fernández-Serrano et al., 2020 [28]<br>Tarancón et al., 2020 [40]<br>Tarancón et al., 2021 [20]<br>Giménez-Sanchis et al., 2021 [42]
Contexts	Jaeger et al., 2019 [37]<br>Di Vita et al., 2020 [39]<br>Verain et al., 2020 [32]<br>Tarancón et al., 2021 [27]
Barriers	Sijtsema et al., 2012 [23]<br>Silvestri et al., 2016 [35]<br>Kasprzak et al., 2021 [41]

Table S3. Ranking of sensory attributes' importance for consumer satisfaction after eating blond/ blood oranges. Importance was scored in a 7-point scale (1-not important at all, 7-very important) by Spanish, Italian, Mexican and Chinese consumers (n=605 in each country). Mean scores are shown in brackets.

Spain		Italy		Mexico		China	
**Blond**	**Blood**	**Blond**	**Blood**	**Blond**	**Blood**	**Blond**	**Blood**
Juicy (6.4) [a]	Juicy (6.2) [a]	Juicy (6.4) [a]	Juicy (6.5) [a]	Juicy (6.8) [a]	Juicy (6.7) [a]	Juicy (6.1) [a]	Juicy (6.2) [a]
Flavour (6.0) [b]	Sweet (5.9) [ab]	Flavour (6.3) [a]	Flavour (6.3) [b]	Sweet (6.4) [b]	Sweet (6.5) [ab]	Sweet (6.1) [ab]	Sweet (6.0) [ab]
Balance (6.0) [b]	Balance (5.8) [ab]	Aromatic (5.8) [b]	Aromatic (6.0) [c]	Balance (6.3) [bc]	Flavour (6.2) [bc]	Unfibrous (6.0) [b]	Aromatic (6.0) [ab]
Sweet (5.8) [c]	Flavour (5.8) [ab]	Balance (5.8) [b]	Sweet (5.8) [c]	Flavour (6.1) [c]	Balance (6.2) [bc]	Aromatic (5.9) [bc]	Unfibrous (6.0) [ab]
Aromatic (5.6) [d]	Aromatic (5.7) [ab]	Sweet (5.7) [b]	Balance (5.8) [c]	Aromatic (5.7) [d]	Aromatic (6.0) [cd]	Flavour (5.9) [bc]	Balance (6.0) [ab]
Unfibrous (5.6) [d]	Colour (5.5) [bc]	Colour (5.0) [c]	Colour (5.3) [d]	Unfibrous (5.7) [d]	Unfibrous (5.8) [d]	Balance (5.9) [bc]	Flavour (6.0) [ab]
Easy Pe (4.7) [e]	Unfibrous (5.3) [c]	Unfibrous (4.5) [d]	Unfibrous (4.6) [e]	Colour (5.4) [e]	Colour (5.8) [d]	NoMessy (5.8) [c]	NoMessy (5.9) [bc]
Seedless (4.6) [e]	Easy Pe (4.7) [d]	Easy Pe (4.4) [d]	Easy Pe (4.4) [e]	Easy Pe (5.0) [f]	Easy Pe (5.3) [e]	Easy Pe (5.7) [c]	Easy Pe (5.9) [bc]
Colour (4.6) [e]	Seedless (4.5) [d]	Seedless (4.0) [e]	Seedless (4.1) [f]	NoMessy (3.9) [g]	Seedless (4.5) [f]	Seedless (5.4) [d]	Seedless (5.7) [c]
NoMessy (3.6) [f]	NoMessy (3.9) [e]	NoMessy (3.7) [f]	NoMessy (3.9) [g]	Seedless (3.8) [g]	NoMessy (4.5) [f]	Colour (5.1) [e]	Colour (5.7) [c]

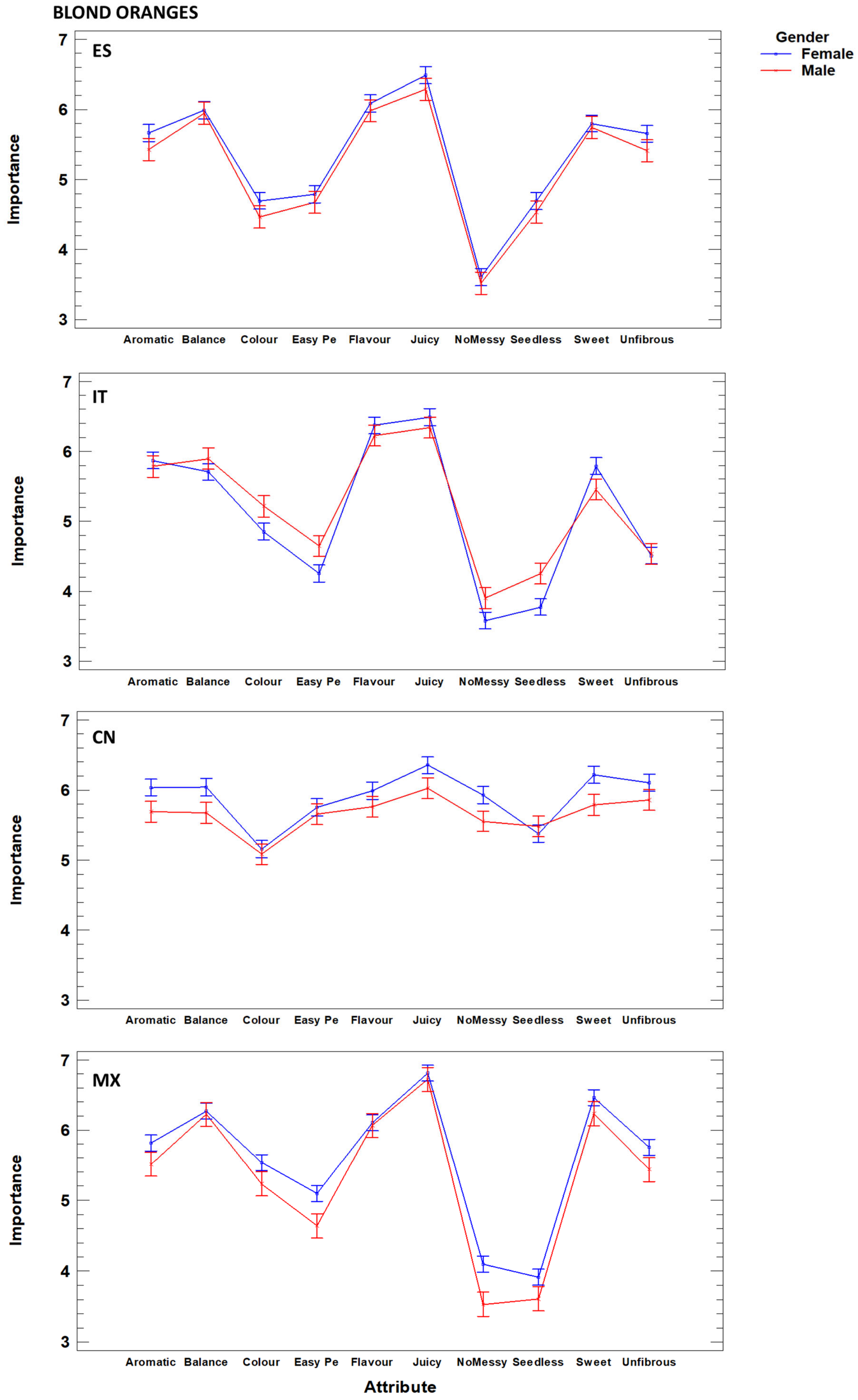

Figure S2. Effect of gender on the importance for satisfaction gave by consumers to different sensory attributes of blond oranges (1-not important at all, 7-very important). ES-Spain, IT-Italy, MX-Mexico, CN-China

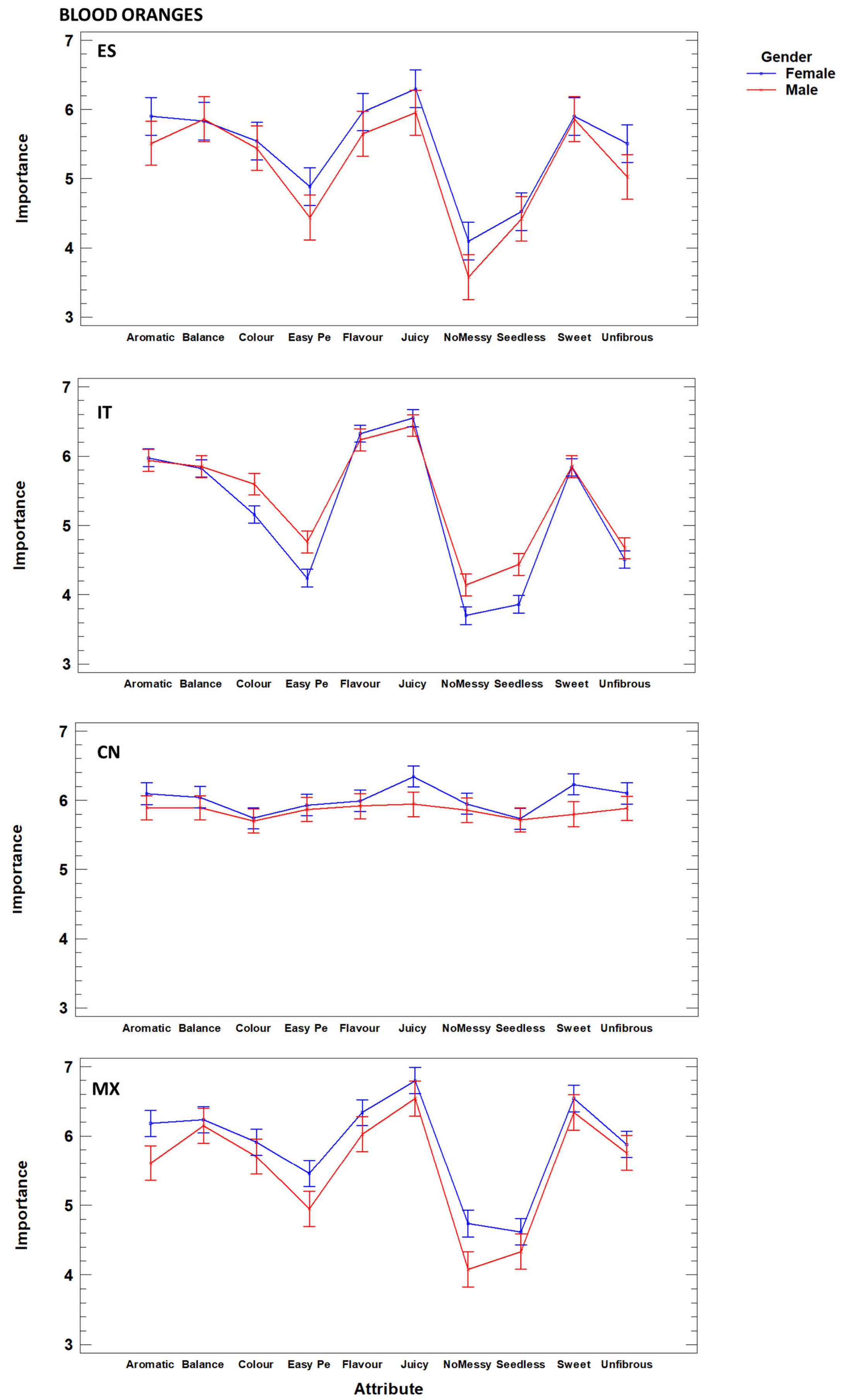

Figure S3. Effect of gender on the importance for satisfaction gave by consumers to different sensory attributes of blood oranges (1-not important at all, 7-very important). ES-Spain, IT-Italy, MX-Mexico, CN-China

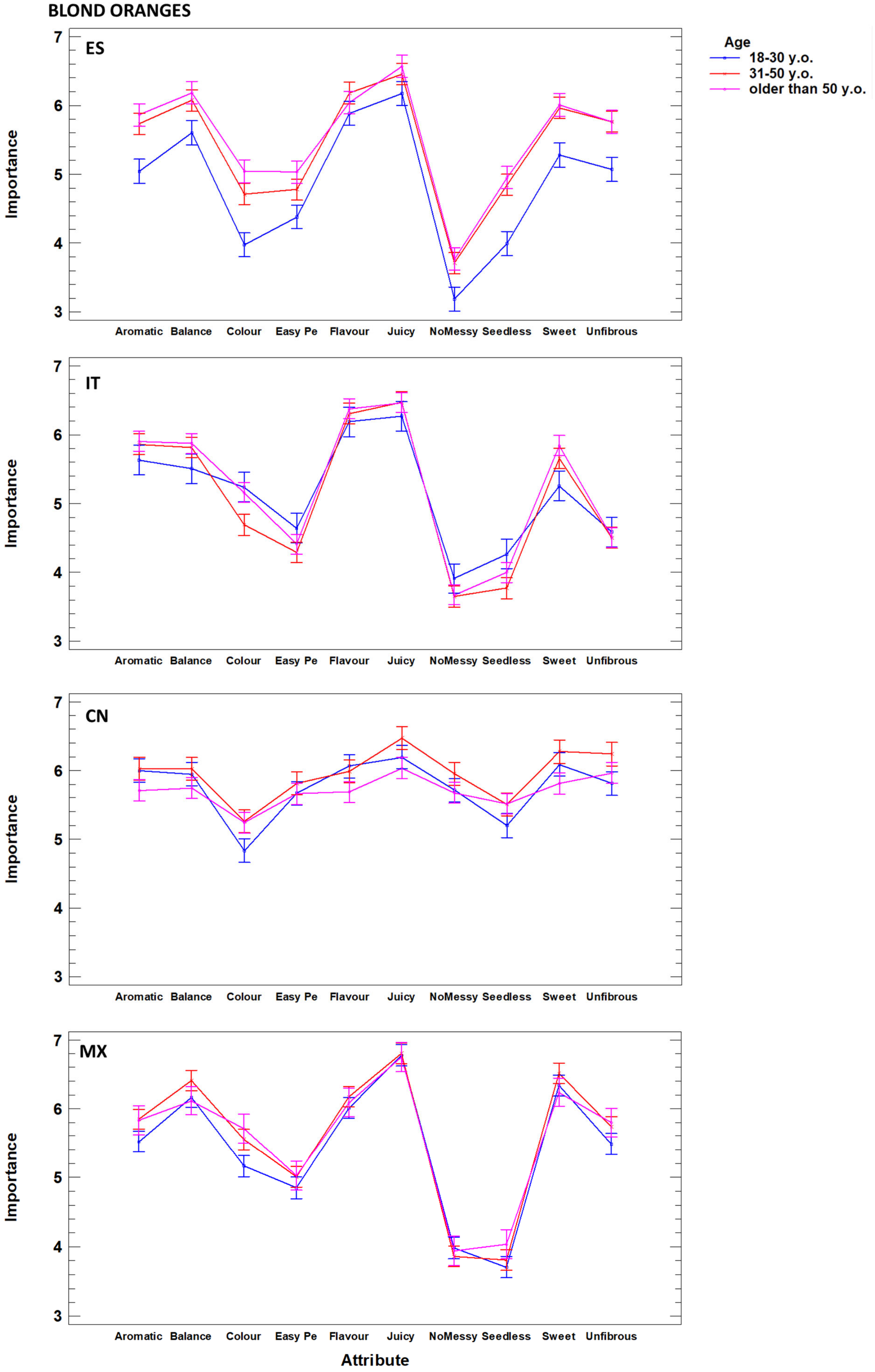

Figure S4. Effect of age range on the importance for satisfaction gave by consumers to different sensory attributes of blond oranges (1-not important at all, 7-very important). ES-Spain, IT-Italy, MX-Mexico, CN-China

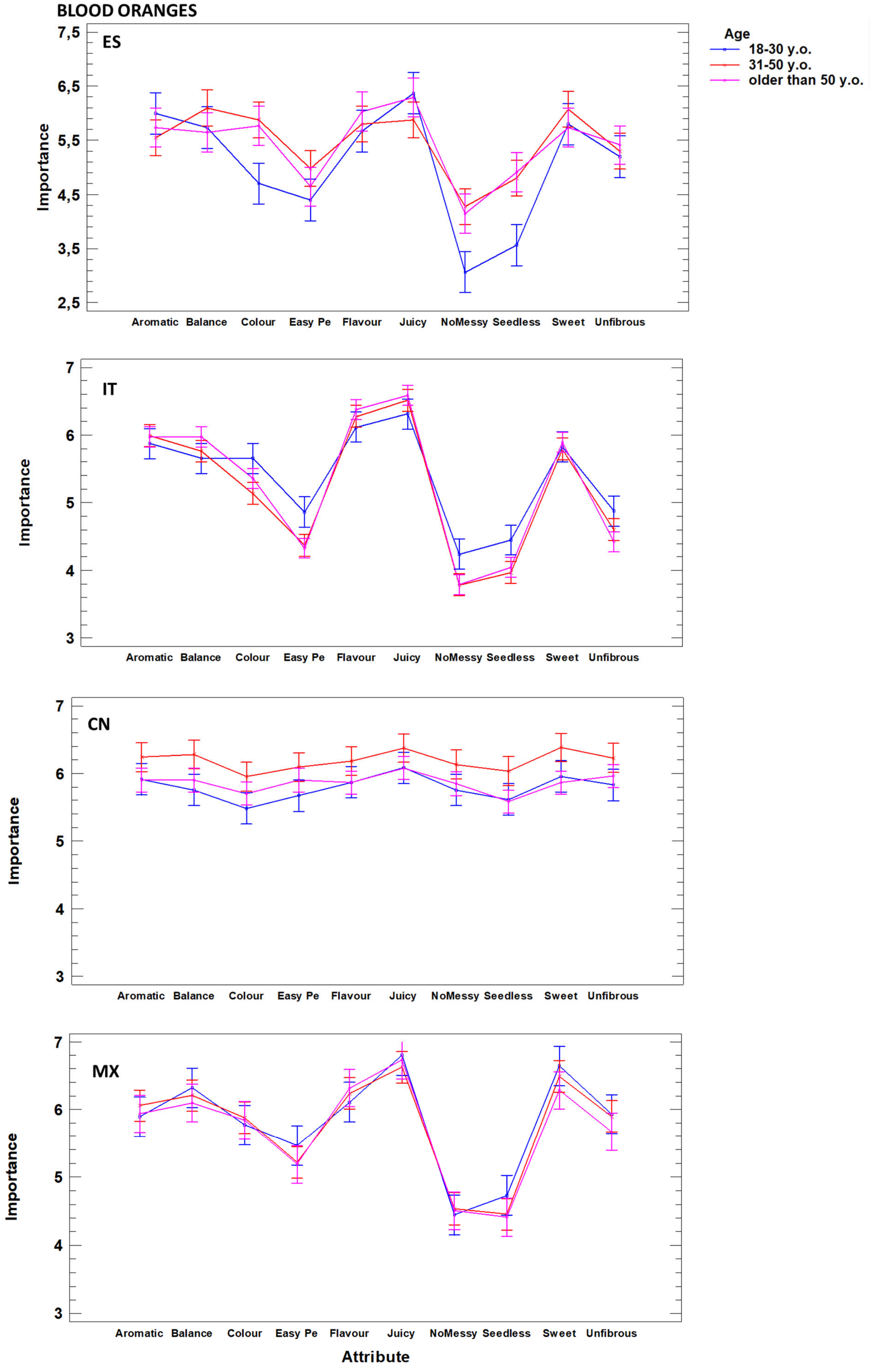

Figure S5. Effect of age range on the importance for satisfaction gave by consumers to different sensory attributes of blood oranges (1-not important at all, 7-very important). ES-Spain, IT-Italy, MX-Mexico, CN-China

Table S4. Percentage of participants (of those who stated consuming each orange type habitually) from each country who selected each consumption context.

Spain		Italy	
**Blond**	**Blood**	**Blond**	**Blood**
Dessert (77.9 %)	Breakfast (54.8 %)	Juice/Smoothy (68.1 %)	Juice/Smoothy (66.7 %)
Juice/Smoothy (70.0 %)	Dessert (51.0 %)	Snack home (63.5 %)	Snack home (64.9 %)
Breakfast (63.6 %)	Snack home (44.2 %)	Dessert (51.8 %)	Dessert (45.8 %)
Snack home (52.7 %)	Juice/Smoothy (26.9 %)	Cooked (40.6 %)	Breakfast (40.7 %)
Improve health (38.5 %)	Snack out home (26.9 %)	Ingredient (40.6 %)	Cooked (31.7 %)
Snack out home (38.1 %)	Improve health (25.0 %)	Breakfast (38.6 %)	Ingredient (31.7 %)
Ingredient (27.0 %)	Child lunch-box (20.2 %)	Snack out home (35.3 %)	Snack out home (31.0 %)
Child lunch-box (24.2 %)	Cocktail (17.3 %)	Improve health (34.2 %)	Improve health (28.1 %)
Cocktail (16.1 %)	Ingredient (15.4 %)	Cocktail (18.7 %)	Cocktail (12.8 %)
Restaurant (15.4 %)	Restaurant (8.7 %)	Child lunch-box (10.6 %)	Child lunch-box (9.0 %)
Weight loss (9.9 %)	Weight loss (7.7 %)	Weight loss (9.5 %)	Weight loss (7.0 %)
Sugar (7.9 %)	Gift (5.8 %)	Salt/chilly powder (4.7 %)	Gift (4.9 %)
Gift (7.5 %)	Sugar (4.8 %)	Sugar (4.2 %)	Salt/chilly powder (4.0 %)
Salt/chilly powder (2.6 %)	Salt/chilly powder (1.0%)	Gift (3.8 %)	Restaurant (3.6 %)
Cooked (1.7 %)	Cooked (1.0%)	Restaurant (3.6 %)	Sugar (2.7 %)
**Mexico**		**China**	
**Blond**	**Blood**	**Blond**	**Blood**
Snack home (75.1 %)	Snack home (66.2 %)	Snack home (71.3 %)	Snack home (65.8 %)
Snack out home (70.3 %)	Breakfast (65.7 %)	Improve health (46.4 %)	Improve health (42.1 %)
Breakfast (64.2 %)	Snack out home (63.3 %)	Snack out home (42.6 %)	Snack out home (39.2 %)
Salt/chilly powder (63.8 %)	Dessert (46.9 %)	Dessert (40.6 %)	Dessert (33.1 %)
Juice/Smoothy (49.6 %)	Salt/chilly powder (46.4 %)	Juice/Smoothy (32.1 %)	Breakfast (32.4 %)
Improve health (47.2 %)	Improve health (45.4 %)	Gift (30.3 %)	Gift (29.1 %)
Dessert (46.5 %)	Juice/Smoothy (37.7 %)	Breakfast (22.4 %)	Juice/Smoothy (25.2 %)
Ingredient (40.0 %)	Cocktail (34.8 %)	Weight loss (22.4 %)	Weight loss (24.5 %)
Cocktail (32.7 %)	Ingredient (31.4 %)	Restaurant (18.6 %)	Restaurant (17.3 %)
Child lunch-box (31.7 %)	Child lunch-box (29.0 %)	Child lunch-box (13.0 %)	Child lunch-box (12.2 %)
Weight loss (14.9 %)	Weight loss (16.4 %)	Ingredient (7.8 %)	Ingredient (8.6 %)
Restaurant (8.3 %)	Restaurant (11.6 %)	Cocktail (4.7 %)	Cocktail (5.0 %)
Gift (8.1 %)	Gift (8.7 %)	Cooked (3.1 %)	Cooked (1.8 %)
Sugar (5.9 %)	Sugar (5.3 %)	Salt/chilly powder (2.2 %)	Salt/chilly powder (1.4 %)
Cooked (3.1 %)	Cooked (3.4 %)	Sugar (1.1 %)	Sugar (0.7 %)

Table S5. Percentage of participants (of those who stated not consuming each orange type habitually)) from each country who selected each consumption barrier.

Spain		Italy	
**Blond**	**Blood**	**Blond**	**Blood**
I prefer other fruits (67.5 %)	I prefer other fruits (58.9 %)	I prefer other fruits (60.0 %)	I prefer other fruits (70.1 %)
I don't know how to prepare it (12.0 %)	I don't like it (17.4 %)	I don't like it (12.0 %)	I don't like it (16.4 %)
I don't like it (9.6 %)	I have never tasted it (13.2 %)	Too expensive (6.0 %)	Too expensive (9.0 %)
Inconvenient (8.4 %)	I don't like the aspect (7.4 %)	I don't like the aspect (4.0 %)	I don't like the aspect (6.0 %)
Too expensive (7.2 %)	Too expensive (6.8 %)	They are not natural (4.0 %)	They are not natural (3.0 %)
They are not natural (3.6 %)	I don't know how to prepare it (1.6 %)	I don't know how to prepare it (2.0 %)	I have never tasted it (2.2 %)
I don't like the aspect (1.2 %)	Inconvenient (0.0 %)	I think they are not safe (2.0 %)	I think they are not safe (0.7 %)
I have never tasted it (0.0 %)	I think they are not safe (0.0 %)	I have never tasted it (0.0 %)	I don't know how to prepare it (0.0 %)
I think they are not safe (0.0 %)	They are not natural (0.0 %)	Inconvenient (0.0 %)	Inconvenient (0.0 %)
**Mexico**		**China**	
**Blond**	**Blood**	**Blond**	**Blood**
I prefer other fruits (61.3 %)	I prefer other fruits (48.0 %)	I prefer other fruits (36.4 %)	I prefer other fruits (31.9 %)
Too expensive (9.7 %)	I have never tasted it (28.0 %)	Too expensive (22.9 %)	Too expensive (30.1 %)
I don't like the aspect (8.1 %)	I don't like the aspect (15.0 %)	I don't like it (21.4 %)	I don't like it (27.9 %)
I think they are not safe (6.5 %)	Too expensive (13.0 %)	Inconvenient (15.7 %)	I have never tasted it (16.8 %)
They are not natural (4.8 %)	They are not natural (11.0 %)	I don't know how to prepare it (11.4 %)	I don't like the aspect (15.5 %)
I don't like it (3.2 %)	I don't know how to prepare it (8.0 %)	They are not natural (10.7 %)	I don't know how to prepare it (13.7 %)
I don't know how to prepare it (3.2 %)	I don't like it (6.0 %)	I don't like the aspect (10.0 %)	They are not natural (11.1 %)
I have never tasted it (0.0 %)	I think they are not safe (5.0 %)	I think they are not safe (9.3 %)	I think they are not safe (6.2 %)
Inconvenient (0.0 %)	Inconvenient (0.0 %)	I have never tasted it (5.7 %)	Inconvenient (0.0 %)

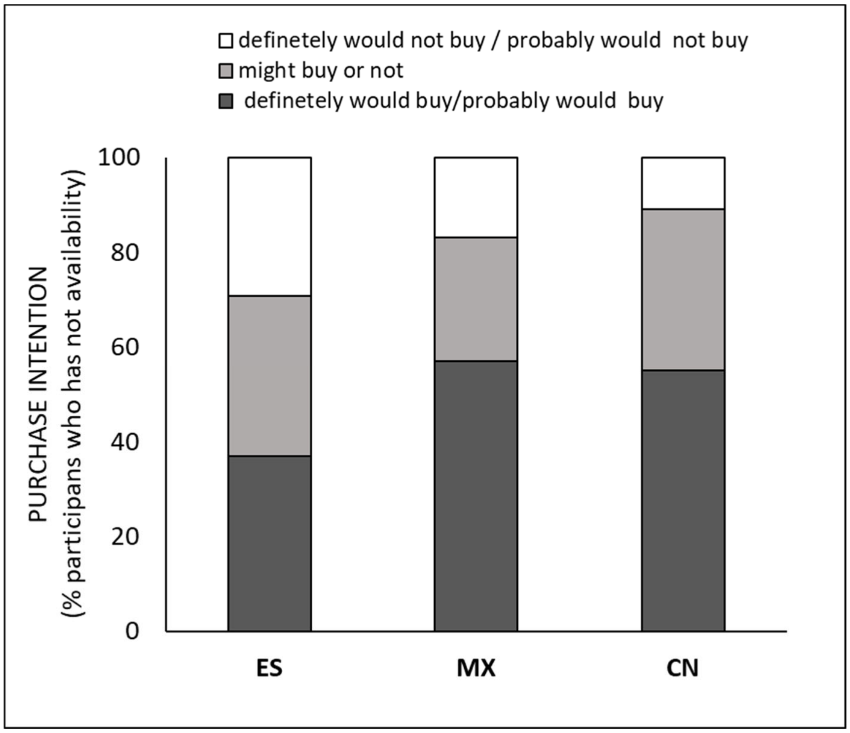

Figure S6. Purchase intention of those consumers for whom blood oranges are not available in their shopping place if oranges were available. ES-Spain, MX-Mexico, CN-China
